# Using a spatial filter and a geographic information system to improve rabies surveillance data.

**DOI:** 10.3201/eid0505.990501

**Published:** 1999

**Authors:** A Curtis

**Affiliations:** Howe/Russell Geoscience Complex, Louisiana State University, Baton Rouge, LA 70803-4105, USA. acurti1@lsu.edu

## Abstract

The design and coordination of antirabies measures (e.g., oral vaccine and disease awareness campaigns) often depend on surveillance data. In Kentucky, health officials are concerned that the raccoon rabies epizootic that has spread throughout the east coast since the late 1970s could enter the state. The quality of surveillance data from Kentucky's 120 counties, however, may not be consistent. This article presents a geographic model that can be used with a geographic information system (GIS) to assess whether a county has a lower number of animals submitted for rabies testing than surrounding counties. This technique can be used as a first step in identifying areas needing improvement in their surveillance scheme. This model is a variant of a spatial filter that uses points within an area of analysis (usually a circle) to estimate the value of a central point. The spatial filter is an easy-to-use method of identifying point patterns, such as clusters or holes, at various geographic scales (county, intraurban), by using the traditional circle as an area of analysis or a GIS to incorporate a political shape (county boundary).


### Using a Spatial Filter and a Geographic Information System to Improve Rabies Surveillance Data

**Andrew Curtis**
Louisiana State University, Baton Rouge, Louisiana, USA

The design and coordination of antirabies measures (e.g., oral vaccine and disease awareness campaigns) often depend on surveillance data. In Kentucky, health officials are concerned that the raccoon rabies epizootic that has spread throughout the east coast since the late 1970s could enter the state. The quality of surveillance data from Kentucky's 120 counties, however, may not be consistent. This article presents a geographic model that can be used with a geographic information system (GIS) to assess whether a county has a lower number of animals submitted for rabies testing than surrounding counties. This technique can be used as a first step in identifying areas needing improvement in their surveillance scheme. This model is a variant of a spatial filter that uses points within an area of analysis (usually a circle) to estimate the value of a central point. The spatial filter is an easy-to-use method of identifying point patterns, such as clusters or holes, at various geographic scales (county, intraurban), by using the traditional circle as an area of analysis or a GIS to incorporate a political shape (county boundary).

Two raccoon rabies epizootics have been spreading from separate sites in the eastern United States. The original site was in Florida in the 1950s (1), the second in West Virginia/ Virginia in 1977 as a result of the importation of rabid raccoons from Florida by hunters (2). In 1994, the two rabies waves met in North Carolina (3). Kentucky has so far not been affected by the epizootic because of geographic barriers—the Appalachian Mountains in the east and the Ohio River to the north of the state.

Raccoon rabies could enter Tennessee and from there move relatively unhindered into the Bluegrass area of central Kentucky (4). Raccoons are the wild animal most frequently submitted for rabies testing in Kentucky (150, 145, and 169 animals in 1995-97, respectively), even though the annual number of positive cases remains low (1 to 2 cases per year). These positive cases appear to occur as a result of spillover infection with skunk rabies, a variant of the virus that differs from the current raccoon rabies epizootic on the eastern seaboard. The high number of raccoons submitted for testing, however, indicates potential for an increase in raccoon rabies similar to that in eastern seaboard states (5-7). In New York State, for example, the number of rabid raccoons increased from 0 in 1985 to >2,000 in 1993.

Surveillance data are useful in coordinating the placement of oral vaccination containment lines to limit the spread of the disease or quickly identify clusters of rabies beyond the containment zone. These data usually are provided by county officials or by the public after animal-human interaction (bite, scratch) or after an encounter with an animal exhibiting rabies symptoms. These data underrepresent the actual extent of the disease, because not all animals with rabies interact with people and not all interactions are reported (5,8,9). The quality of surveillance data varies among counties for several reasons (10, unpub. data). According to multivariate analysis, the number of animals submitted for testing was positively correlated with the number of people living in the county and negatively correlated with distance to the state testing facility in Frankfort (unpub. data).

Address for correspondence: Andrew Curtis, E110 Howe/ Russell Geoscience Complex, Louisiana State University, Baton Rouge, LA 70803-4105, USA; fax: 225-388-4420; e-mail: acurti1@lsu.edu.

We describe a method that can improve the quality of surveillance data by identifying counties that submit lower numbers of animals for testing than their surrounding counties. This method is a variation of a spatial filter that is calculated within a geographic information system (GIS).

### Using a Spatial Filter in a GIS

When counties that submit few animals for rabies testing are surrounded by counties with high numbers of submissions, underreporting is suspected. Different methods of data analysis can produce different visual (mapped) results. Conclusions, and resulting policy actions, therefore may vary according to the method chosen.

Figure 1 shows three maps that can be generated from the rabies surveillance data: actual numbers of submissions; standardized submissions (submissions per 10,000 human population); and the mapped residuals of a regression of submissions compared with the human population. Results can also vary according to the classification scheme chosen, that is, the quantity and range of data assigned to each color category on the map. The size and shape of the area investigated (county units, zip codes, census tracts) may also produce different results, depending on the chosen political unit—an effect known in geographic research as the Modifiable Area Unit Problem (11).

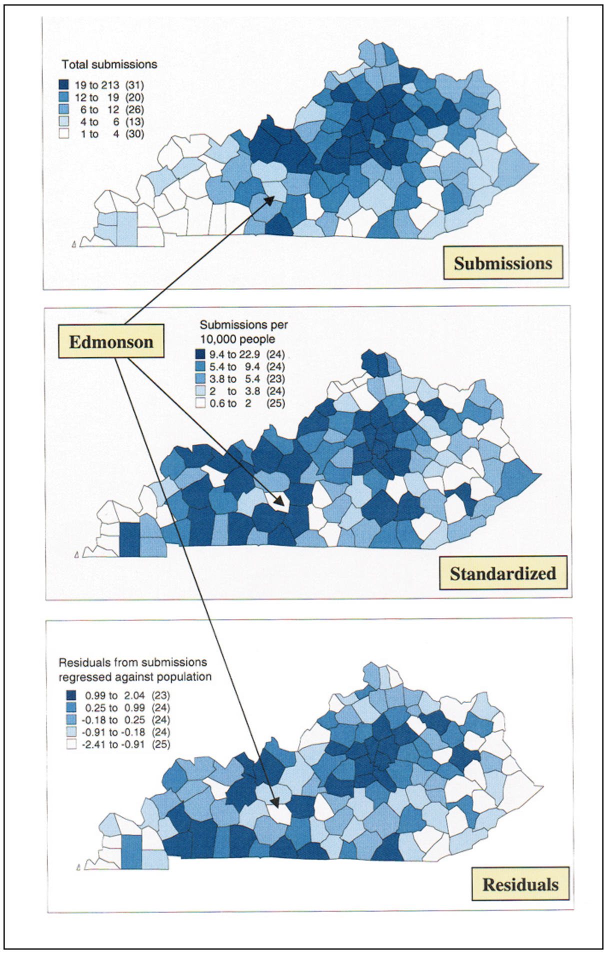

Figure 1. Comparison of data analysis for identifying counties with low submission rates.

Spatial filter models are methods of exploratory spatial analysis that smooth point data, allowing values for central data points to be calculated. The traditional spatial filter technique, as applied to medical research, involves placing a grid over the investigated area, with the intersections of the grid lines providing the centers of a series of overlapping circles. Rates are then calculated for these circles to give a continuous surface. This approach has the potential to better replicate distribution of a disease, because diseases do not usually follow political boundaries. An important aspect of the spatial filter is the size of the circle, i.e., the area over which the analysis is performed, which can affect the analysis result (12).

### Applying the Spatial Filter to the Number of Animals Submitted for Rabies Testing in Kentucky

In this Kentucky example, the spatial filter is the county to be investigated and a sphere of influence around it. GIS modifies the shape of the filter to include a buffer that follows the exact shape of the county. The size of the filter is the extension of the buffer beyond the county boundary. The total number of points (numbers of animals submitted for rabies testing) that fall within the filter is then randomly distributed across the area, and this randomization is repeated 100 times. If the number of randomly generated points in the investigated county is lower than the actual number of surveillance points in fewer than 5 of the 100 random runs, there is a 95% chance that a significantly low frequency (number of animals submitted for testing) was reported from that county.

### Applying the Spatial Filter to Surveillance Data in Kentucky

The different maps of animals submitted for rabies testing in Kentucky for 1997 (Figure 1) confirm that several counties appear to submit low numbers of animals. For example, one county we investigated, Edmonson, although appearing to be a 'hole' in both the map of raw animal submission numbers and the map of residuals generated by the regression of submissions compared with the human population, does not appear to have underreported when the data are standardized by the human population. A benefit of the spatial filter is that it can be applied to any variable with a spatial location. This analysis depends on an initial visual interpretation because the data are aggregated at the county level.

In 1997, two animals were submitted for testing from Edmonson County. The surrounding five counties submitted 98 animals for testing, for a total of 100 submissions from the six-county area (Figure 2a). The first step is to randomly distribute all submissions across the county of origin (Figure 2b). Using the actual origin of the animal would improve the analysis, as the more traditional spatial filter method could be used; however, this information is not available from the submissions record. The second step is to layer a grid of coordinates across the six-county area (Figure 2c). This grid consists of closely packed coordinates onto which the submissions for each county can be randomly assigned. The spatial filter is then centered on Edmonson County and a buffer of 5 km is drawn. The buffer size of 5 km provides a standard buffer that could be used for any county, and which would, on average, match the area of an investigated county. The randomly distributed animal submissions for each of the surrounding five counties are then displayed to see how many animals were submitted for testing from the buffered area (Figure 2d). The simulation can be repeated several times, and a histogram can be used to identify the most frequent number of animal submissions from the buffer being chosen for the filter analysis. For this example, 12 animals were allocated to the area of Edmonson County and its surrounding 5-km buffer (two from Edmonson County and 10 from the buffer area). Within a GIS these 12 points are randomly redistributed across the area of Edmonson County plus buffer. This simulation procedure is repeated 100 times (Figure 2e displays 5, 6, and 8 submissions falling on the central county). The number of points falling just within Edmonson County for each of the random runs was recorded. In 100 runs, the fewest number of points (animal submissions) allocated to Edmonson County was three. Thus, the number of submissions is significantly low compared with those from the surrounding counties. Our analysis did not replicate the number of submissions from Edmonson County in 1997 in any of the 100 simulations generated from a data distribution that matched the surveillance data of 1997.

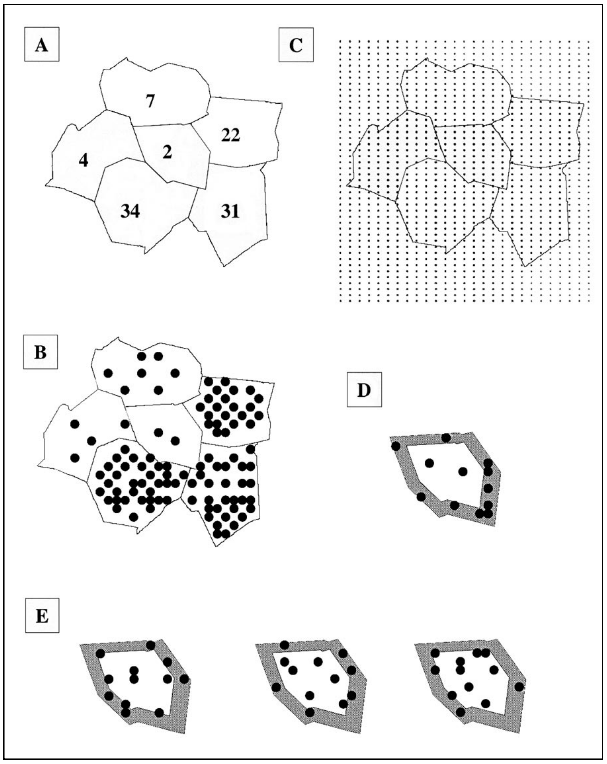

Figure 2. Application of spatial filter with a geographic information system.

The lower count from Edmonson County may result from a smaller human population, more limited animal habitat, or fewer human-animal interactions. The landscape of the buffer may also differ from that of the surrounding counties, making it poor habitat for raccoons. This technique identifies 'holes' in data, prompting further investigation for causes.

### Improvements to the Filter

The most important improvement in using the spatial filter technique is that current submission records should start to contain precise spatial locations. Surveillance data from other states contain a spatial location, such as the distance from a road intersection (6), obtained in postrabies confirmation interview. A Global Positioning System, or even a systematic method to locate submissions according to a paper map reference system, would allow the spatial filter to be calculated as a series of overlapping circles that do not depend on political boundaries.

The technique presented in this article provides an investigator, such as a state health official, with a quick and accurate method of identifying statistically significant data holes in a surface of animals submitted for rabies testing. A decision can then be made as to whether the 'hole' is expected or needs further investigation. This technique can also be applied to any point data surface where the identification of a 'hole' is important, e.g., if one part of a suburb has fewer Lyme disease cases than its surrounding areas.

### Acknowledgments

The author thanks M.B. Auslander for his continued support and R. Mitchelson for his critical review of this manuscript.

This work was funded by a research grant from Morehead State University.

Dr. Curtis is an instructor at the Department of Geography and Anthropology at Louisiana State University. His research interests include developing spatial analysis within a GIS environment, with a particular emphasis on medical data.

### References

1. Bigler WJ, McLean RG, Trevino HA. Epizootiologic aspects of raccoon rabies in Florida. Am J Epidemiol 1973;98:326-35.
2. Jenkins SR, Winkler WG. Descriptive epidemiology from an epizootic of raccoon rabies in the middle Atlantic states, 1982-1983. Am J Epidemiol 1987;126:429-37.
3. Krebs JW, Strine TW, Smith JS, Rupprecht CE, Childs JE. Rabies surveillance in the United States during 1994. J Am Vet Med Assoc 1995;207:1562-75.
4. Taylor L. Raccoons expected to spread risk in Ky. Lexington Herald-Leader May 25, 1998; Business Monday p. 11.
5. Wilson ML, Bretsky PM, Cooper GH Jr, Egbertson SH, Van Kruiningen HJ, Cartter ML. Emergence of raccoon rabies in Connecticut, 1991-1994: spatial and temporal characteristics of animal infection and human contact. Am J Trop Med Hyg 1997;57:457-63.
6. Hubbard DR. A descriptive epidemiological study of raccoon rabies in a rural environment. J Wildl Dis 1985;21:105-10.
7. Beck A, Felser S, Glickman L. An epizootic of rabies in Maryland, 1982-84. Am J Public Health 1987;77:42-44.
8. Krebs JW, Wilson ML, Childs JE. Rabies-epidemiology, prevention, and future research. Journal of Mammalogy 1985;76:681-94.
9. Smith JS. New aspects of rabies with emphasis on epidemiology, diagnosis, and prevention of the disease in the United States. Clin Microbiol Rev 1996;Apr:166-176.
10. Heidt G, Ferguson D, Lammers J. A profile of reported skunk rabies in Arkansas: 1977-1979. J Wildl Dis 1982;18:269-77.
11. Curtis A, MacPherson AD. The zone definition problem in survey research: an empirical example from New York State. The Professional Geographer 1996;48:310-20.
12. Scott DW. Multivariate density estimation: theory, practice and visualization. New York: J. Wiley; 1992.

## References

[PDF_00282] Beck A. M., Felser S. R., Glickman L. T. (1987). An epizootic of rabies in Maryland, 1982-84.. Am J Public Health.

[PDF_00262] Bigler W. J., McLean R. G., Trevino H. A. (1973). Epizootiologic aspects of raccoon rabies in Florida.. Am J Epidemiol.

[PDF_00290] Heidt G. A., Ferguson D. V., Lammers J. (1982). A profile of reported skunk rabies in Arkansas: 1977-1979.. J Wildl Dis.

[PDF_00279] Hubbard D. R. (1985). A descriptive epidemiological study of raccoon rabies in a rural environment.. J Wildl Dis.

[PDF_00265] Jenkins S. R., Winkler W. G. (1987). Descriptive epidemiology from an epizootic of raccoon rabies in the Middle Atlantic States, 1982-1983.. Am J Epidemiol.

[PDF_00268] Krebs J. W., Strine T. W., Smith J. S., Rupprecht C. E., Childs J. E. (1995). Rabies surveillance in the United States during 1994.. J Am Vet Med Assoc.

[PDF_00274] Wilson M. L., Bretsky P. M., Cooper G. H., Egbertson S. H., Van Kruiningen H. J., Cartter M. L. (1997). Emergence of raccoon rabies in Connecticut, 1991-1994: spatial and temporal characteristics of animal infection and human contact.. Am J Trop Med Hyg.

